# 

**Published:** 1892-04

**Authors:** 


					﻿Vol. XII.
APRIL, 1892.
No. 4?
THE
pOMEEOPATHlC pHY^lClAfl,
A Monthly Journal of Homoeopathic Materia Medica
and Clinical Medicine.
“ He is a freeman whom truth makes free, and all are slaves beside."
“Seek the Truth: come whence it may, cost what it will.”
EDITED AND PUBLISHED BY
WALTER M. JAMES, M. D.
PHILADELPHIA :
No. 1125 SPRUCK STREET.
LONDON:
ALFRED HEATH & CO., Sole Agents for Great Britain,
114 Ebury Street, Eaton Square.
^•Jtnrly Subscription, $2.50. invariably in advance. Single nsimbt»s, k5 rts.
Entered at the Philadelphia Peet-Offlee ae 8eoond-Clean Mail Matter.
THIS NUMBER, PRICE 60 CTS.
				

